# Metabolic Glucose Status and Pituitary Pathology Portend Therapeutic Outcomes in Acromegaly

**DOI:** 10.1371/journal.pone.0073543

**Published:** 2013-09-09

**Authors:** Sonia Cheng, Rany Al-Agha, Paula B. Araujo, Omar Serri, Sylvia L. Asa, Shereen Ezzat

**Affiliations:** 1 Department of Medicine, University Health Network, Toronto, Ontario, Canada; 2 Department of Pathology, University Health Network, Toronto, Ontario, Canada; 3 Department of Medicine, University of Montreal, Montreal, Canada; University of Cordoba, Spain

## Abstract

**Introduction:**

Acromegaly is frequently associated with impaired glucose tolerance and/or diabetes. To evaluate the relationship between glucose metabolism and acromegaly disease, we evaluated 269 consecutive patients from two referral centres.

**Methods:**

Clinical presentation, pituitary tumor size and invasiveness, and pituitary pathology were captured in a dedicated database.

**Results:**

131 women and 138 men with a mean age of 53.8 years were included. Of these, 201 (74.7%) presented with a macroadenoma and 18 (6.7%) with a microadenoma. Radiographic invasion was present in 91 cases (33.8%). Mean tumor diameter was 1.86 cm (0.2–4.6). Pituitary histopathologic findings revealed pure GH-producing somatotroph adenomas (SA) in 147 patients, prolactin-production by mixed lactotroph (LA) and SA or mammosomatotroph adenoma (MSA) in 46 [22.4%], acidophil stem cell adenoma in 6 [2.9%], and other diagnoses in 6 [2.9%]. Medical treatment included octreotide in 96 [36.9%] and in combination with pegvisomant or dopamine agonists in 63 [24.2%]. Nearly 80% of patients achieved IGF-1 normalization. Importantly, patients with pure somatotroph adenomas were significantly more likely to present with abnormal glucose metabolism [48.7%] than those with mixed adenomas [9.7%] [p<0.001] independent of GH/IGF-1 levels or tumor invasiveness. Abnormal glucose metabolism and pituitary pathology also remained linked following IGF-1 normalization. Moreover patients with pure SA and abnormal glucose metabolism were significantly (p<0.001) less likely to achieve disease remission despite the same therapeutic strategies. Conversely, patients with mixed adenomas were more likely (OR: 2.766 (95% CI: 1.490–5.136) to achieve disease remission.

**Conclusions:**

Patients with pure somatotroph adenomas are more likely than those with mixed adenomas to exhibit abnormal glucose metabolism.

## Introduction

Acromegaly is a disease most frequently resulting from overproduction of growth hormone (GH) by a pituitary adenoma. Aside from the effects of GH and IGF-1 directly on peripheral tissues, morbidity and mortality of patients with this disease is strongly associated with diabetes mellitus, hypertension, and cardiovascular disease [1], [2]. Poorly controlled acromegaly is associated with multiple co-morbidities, the most relevant, being cardiovascular, respiratory, rheumatologic, neuropsychiatric, neoplastic and metabolic [3]–[6]. In particular, cardiovascular disease [5], occurring in the form of hypertrophic cardiomyopathy and ischemic heart disease as a consequence of predisposing metabolic complications, accounts for most of the increased mortality in more than 60% of acromegalic patients [7], [8].

Metabolic disturbances including hyperinsulinemia and dyslipidemia are associated with hepatic and peripheral insulin resistance and improve only partially with surgical resection and somatostatin analog therapy [9], [10]. More recently, tighter degrees of acromegaly control using a GH antagonist [11]–[13] appear to favourably impact glucose tolerance. The factors underlying this response are yet to be determined.

Clinical experience with acromegaly management identifies at least two broad groups of patients, those who exhibit an excellent response to therapeutic modalities and those who remain grossly symptomatic with several co-morbidities despite multimodal therapies [14], [15]. Patient characteristics, such as age, have been associated with metabolic complications [16] whereas others, such as gender, have been excluded as determinants of disease prognosis. In addition, specific features of pituitary adenoma pathology have been implicated in therapeutic responsiveness [15].

The aim of this study was to evaluate acromegaly treatment outcomes as they relate to presenting metabolic status and pituitary tumor pathology in two referral centres.

## Patients and Methods

### Patients

We examined 127 consecutive cases of acromegaly from the Endocrine Oncology Clinic, University Health Network, Toronto, Canada. We registered patient age, gender, clinical symptoms upon presentation, pituitary tumor size and invasiveness beyond the sella on MR imaging (MRI). The second cohort included 142 cases from the Centre Hospitalier de l’Université de Montréal (CHUM), a referral centre from Montreal, Canada. The same clinico-pathologic, imaging and biochemical data were also retrospectively registered.

From both referral centres, we collected patient age, gender, presenting clinical symptoms, pituitary tumor size, and invasiveness on MR imaging (MRI). The study was approved by the UHN and the CHUM Research Ethics Boards. Written consent was provided for patient information to be stored and used for research purposes.

### Pituitary Histopathology

We obtained all available histopathology records and registered pathology diagnosis as well as pathology subtype. Tumors with prolactin (PRL) content were classified as polymorphous mixed somatotroph/lactotroph adenomas or monomorphous mammosomatotroph adenomas (MSA) or acidophil stem cell adenomas (ASCA); these were classified together in a distinct group from pure GH-producing somatotroph adenomas (SA). Pure somatotroph adenomas (SA) were further subdivided into sparsely granulated (SG) and densely granulated (DG) adenomas according to currently accepted diagnostic criteria [17].

### Metabolic Testing and Diagnostic Criteria

Patients underwent glucose tolerance testing using a 75 grams glucose challenge with concomitant measurement of glucose at baseline (G0) and at 120 minutes (G120) as well as basal growth hormone (bGH), GH at 120 minutes (GH120), nadir GH (nGH) and basal IGF-1 (bIGF-1). We also registered IGF-1 at the last visit and according to age and gender determined IGF-1 normalization. From the G0 and G120 values we defined diabetes (DM) or impaired glucose tolerance (IGT), according to the latest 2011 ADA criteria.

Basal GH (bGH) was considered normal if <2.5 µg/L and hyper-somatotropinemia (hyperGH) if >2.5 µg/L. To avoid confusion over changing assays and diagnostic criteria overtime, nadir GH was considered normal if <1 µg/L and hyperGH if >1 µg/L. Basal prolactin was considered normal if <30 µg/L, moderate hyper-prolactinemia (hyperPRL) if >30 and <75 µg/L and severe hyperPRL >75 µg/L. For some analyses moderate and severe hyperPRL were grouped as hyperPRL as indicated.

### Therapeutic Measures

All therapeutic measures directed towards the management of acromegaly were recorded and grouped by strategy. These included: 1) medical only, 2) surgical only, 3) surgical and medical and 4) surgical, medical, and radiotherapy, we considered for analysis the largest two: 1) surgery alone, and 2) surgery and medical treatment. Patients treated medically were divided into those receiving octreotide alone and those treated with all other medications, including combinations of octreotide with pegvisomant or dopamine agonists.

### Statistical Analyses

Descriptors were reported with means and standard deviation where appropriate, as well as proportions and percentages. For group analyses, text, tables and graphs reflect percentages for each group in square brackets [ ] or in round brackets ( ) for the entire cohort as indicated. All cases were initially included and for each sub-analysis only cases with complete relevant information were selected. The number of cases included per group [N] and subgroup (n) is specified for each analysis. According to each variable distribution, differences in means or between groups were analysed with Student’s t or Mann-Whitney U and differences in proportions using Z, X2 and appropriate post-hoc testing. For risk estimates, we calculated risk ratios and odds ratios with their respective 95% confidence intervals (95% CI). Statistical significance was considered achieved at a level of p<0.05.

## Results

### Patients

A total of 269 patients from the two referral centers, 131 (48.7%) women and 138 (51.3%) men with confirmed acromegaly were included for analysis. Presenting demographics and co-morbidities are depicted in Table 1. Two hundred and one (74.7% of total) presented with macroadenomas and 91 (33.8% of total) were considered to have invasive tumors on MRI [Table 1]. Most patients (260) 96.6% underwent pituitary surgery; combined surgery and medical treatment was the most frequent therapeutic strategy (116 patients 43.1%) followed by pituitary surgery alone (99 patients 36.8%) and combined with radiotherapy (45, 16.7%). One patient refused treatment. The main medical treatment after surgery was octreotide alone (96 [36.9% of 260]) followed by combinations of octreotide with pegvisomant or a dopamine agonist (63 [24.2%]) [Table 2]. Another group of patients received only medical treatment in the form of octreotide without surgery. However, as this group was too small (N = 8), they were not included in subsequent analyses. Baseline IGF-1 was 835.2 µg/L (34.4) [Mean (SE)] for macroadenomas compared with 678.3 µg/L (38.2) for those with microadenomas (p = 0.044). IGF-1 levels showed no significant difference between pure SA and mixed adenomas either at baseline or at last visit. Pre-surgical IGF-1 showed no significant difference according to glucose metabolism and tumor size when assessing IGF-1 normalization [Figure S1–B and C].

**Table 1:** Patients and tumor characteristics.

		N	Valid %
**Gender**	**Female**	131	48.7
	**Male**	138	51.3
**Diabetes (previous diagnosis)**	**No**	102	80.3
	**Yes**	25	19.6
**Hypertension**	**No**	128	57.9
	**Yes**	93	42.1
**Obstructive sleep apnea**	**No**	200	74.3
	**Yes**	69	25.7
**Other cancers**	**No**	201	74.7
	**Yes**	24	8.9
**Osteoarthritis**	**No**	83	65.4
	**Yes**	44	34.6
**Glucose metabolism**	**Normal**	152	56.5
	**Abnormal**	73	27.1
	**Missing**	44	16.4
**Invasion (MRI)**	**Yes**	91	33.8
	**No**	178	66.2
**Pituitary tumor size**	**<1 cm**	46	18.6
	**1–4 cm**	201	81.4
**Pituitary pathology**	**SA**	147	54.6
	**Mixed**	46	17.1
	**ASCA**	6	2.2
**Pathology subtype**	**SG**	70	26.0
	**DG**	49	18.2
**IGF-1 normalization**	**No**	51	21.5
	**Yes**	186	78.5

SA = somatotroph adenoma; ASCA = acidophil stem-cell adenoma, SG = sparsely granulated, DG: densely granulated. Invasiveness was based on clear parasellar extension on MRI.

**Table 2:** Therapeutic strategies.

		N	Valid %
**Pituitary surgery**	**Yes**	259	96.3
	**No**	10	3.7
**Preoperative medical therapy**	**Yes**	28	10.4
	**No**	241	89.6
**Therapeutic strategy**	**Medical only**	8	3.0
	**Surgical only**	99	36.8
	**Surgical+medical**	116	43.1
	**Any+RT**	45	16.7
**Postoperative medical treatment**	**None**	67	29.6
	**LAR alone**	96	35.7
	**All other meds**	63	23.4

RT = radiotherapy; LAR = long-acting octreotide.

GH levels at presentation showed a corresponding pattern. Random GH (rGH) of 38.3 µg/L (5.4) was noted for those with macroadenomas vs. 10.5 µg/L (1.5) for microadenomas (p<0.001). Similarly, nadir GH (nGH) levels during the diagnostic OGTT were 19.3 µg/L (3.1) for macroadenomas and 6.6 µg/L (5.8) for microadenomas (p<0.001). However, no significant differences were noted between presenting GH levels and glucose metabolism status or with IGF-1 normalization [Figure S2–D, E].

### IGF-1 Normalization

Two hundred and thirty seven patients had information to assess IGF-1 normalization at last visit. One hundred and eighty six [78.5%] patients achieved IGF-1 normalization at last visit and 51 patients [21.5%] continued to experience elevated IGF-1 levels. To gain insights into prognostic features that may portend IGF-1 responses, we examined clinical, biochemical, radiographic, and histopathologic characteristics.

Pituitary histopathologic diagnoses were evaluable for 205 cases: there were 147/205 [71.7%] pure SA, 46/205 [22.4%] mixed adenomas, 6/205 [2.9%] ASCA, and 6/205 [2.9%] with other diagnoses.

#### Tumor characteristics

**Figure 1:**
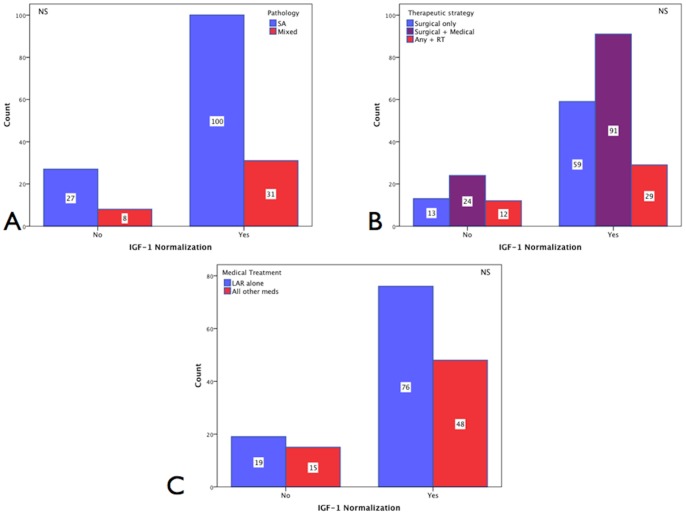
IGF-1 normalization. A. Pathology. IGF-1 normalization was achieved in 100 [60.2%] of those with pure SA and 31 [18.7%] cases of those with mixed tumors vs. 27 [16.3%] and 8 [4.8%] not normalizing **[N = 166]** (NS). **B. Therapeutic strategy.** IGF-1 normalization was achieved by surgery in 59 [25.9%] cases, 91 [39.9%] by surgery+medical therapy, 29 [12.7%] by the addition of radiotherapy vs. 13 [5.5%], 24 [10.1%] and 12 [4.46%] cases in each group not normalizing IGF-1 **[N = 228]** (NS). **C. Medical treatment.** IGF-1 normalization in the medically treated group was achieved in 76 [48.1%] cases using LAR alone, and 48 [30.4%] with other medications vs. 19 [12.0%] and 15 [9.5%] not achieving normalization **[N = 158]** (NS).

From 215 cases with evaluable information on pituitary tumor size and IGF-1 levels, patients with macro-adenomas achieved IGF-1 normalization in 138 [77.5% of 178] instances and those with micro-adenomas in 33 [89.2% of 37] cases (Table 1). Other tumor characteristics such as tumor invasion on MRI and histopathologic diagnoses, including pituitary pathology subtyping, showed no significant differences with IGF-1 normalization (Table 3 and Figure 1–A).

**Table 3:** IGF-1 normalization according to pituitary tumor characteristics.

		Normal IGF-1 at last visit			
		Yes: N (%)	No: N (%)	Total	p
**Tumor size**	**<1 cm**	33 (89.2)	4 (10.8)	37	p = 0.058
	**>1 cm**	138 (77.5)	40 (22.5)	178	
**Invasion (MRI)**	**No**	121 (79.1)	32 (20.9)	153	NS
	**Yes**	65 (77.4)	19 (22.6)	84	
**Pathology**	**SA**	100 (78.7)	27 (21.3)	127	NS
	**Mixed**	31 (79.5)	8 (20.5)	39	
	**ASCA**	2 (66.7)	1 (33.3)	3	
**Pathology subtype**	**SG**	50 (79.4)	13 (20.6)	63	NS
	**DG**	37 (82.2)	8 (17.7)	45	

SA = somatotroph adenoma; ASCA: acidophil stem-cell adenoma, SG: sparsely granulated, DG: densely granulated.

#### IGF-1 normalization according to therapeutic strategy

From 237 therapeutically evaluable cases IGF-1 normalization was achieved by surgery alone in 59/72 [81.9%] cases, in 91/115 [79.1%] cases by combined surgery and medical therapy, and in 29/41 [70.7%] cases by the addition of radiotherapy. The corresponding proportions for those whose IGF-1 did not normalize were: 13/72 [18.0%] surgery alone, 24/115 [20.8%] surgery and medical therapy, and 12/41 [29.2%] radiotherapy cases (NS) [Figure 1–B]. For patients receiving postoperative medical therapy **[N = 158]**, IGF-1 normalization was achieved in 76/95 [48.1%] of cases using octreotide alone, and in 48/63 [30.4%] of those receiving additional medications (see Patients and Methods), whereas 19/95 [12%] and 15/63 [9.5%] in each group did not achieve IGF-1 normalization (NS) [Figure 1–C].

### Glucose Metabolism Status at Presentation

#### Glucose status according to pituitary tumor size

**Figure 2:**
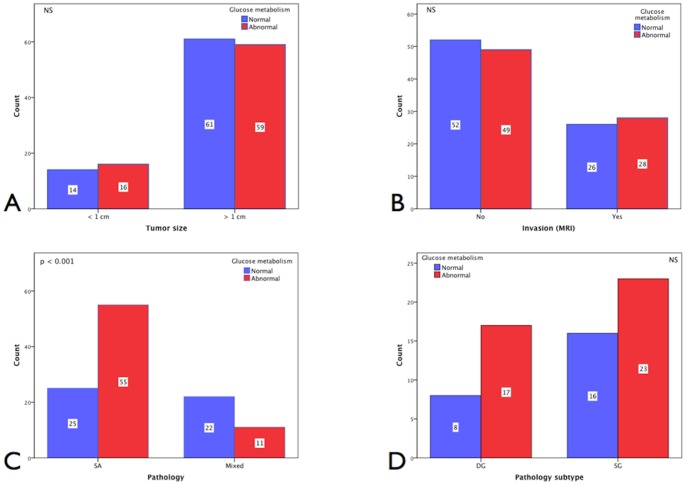
Baseline glucose metabolism status and pituitary tumor characteristics. **A**. Glucose metabolism status at presentation according to tumor size. Fourteen [9.3%] of patients with microadenomas and 61 [40.7%] with macroadenomas presented with normal glucose metabolism vs. 16 [10.7%] with microadenomas and 59 [39.3%] with macroadenomas revealed abnormal glucose metabolism at presentation **[N = 150]** (NS). **B.** Glucose metabolism status according to invasion (MRI) revealed that 52 [33.5%] non-invasive and 26 [16.8%] of invasive cases presented with normal glucose metabolism vs. 49 [31.6%] non-invasive and 28 [18.1%] invasive with abnormal metabolism **[N = 155]** (NS). **C.** Glucose metabolism status according to pituitary tumor pathology revealed that 25 [22.1%] of patients with pure SA and 22 [19.5%] with mixed adenomas presented with normal glucose metabolism compared to 55 [48.7%] with pure SA and 11 [9.7%] with mixed adenomas who presented with abnormal glucose metabolism **[N = 113]** (p<0.001). **D.** Glucose metabolism status according to pituitary pathology subtype revealed that 8 [12.5%] DG and 16 [25.0%] SG presented with normal glucose metabolism vs. 17 [26.6%] DG and 23 [35.9%] SG already had abnormal glucose metabolism **[N = 64]** (NS).

From 150 cases with evaluable pituitary tumor size and glucose metabolism data, 14 [9.3%] patients with microadenomas and 61 [40.7%] with macroadenomas presented with normal glucose metabolism by OGTT as opposed to 16 [10.7%] with microadenomas and 59 [39.3%] with macroadenomas who exhibited abnormal glucose metabolism at presentation **[N = 150]** (NS) [Figure 2–A]. Analyses based on tumor invasion on MRI **[N = 155]** revealed that 52 [33.5%] non-invasive and 26 [16.8%] invasive cases had normal glucose metabolism at presentation whereas 49 [31.6%] non-invasive and 28 [18.1%] invasive presented with abnormal metabolism (NS) [Figure 2–B]. These findings suggested that pituitary tumor size or invasiveness were not prognostic features strictly associated with impaired glucose tolerance.

#### Glucose status according to pituitary tumor pathology

Pituitary histopathology diagnosis of either a mixed or a pure SA: **[N = 113]** revealed that 25 [22.1%] of patients with pure SA and 22 [19.5%] with mixed adenomas presented with normal glucose metabolism. In marked contrast, nearly half of patients [55, 48.7%] with pure SA compared to only 11 [9.7%] with mixed adenomas presented with abnormal glucose metabolism at diagnosis (p<0.001) [Figure 2–C]. This association was independent of tumor sub-typing [Figure 2–D]. These findings suggested that glucose metabolism at the time of acromegaly diagnosis maybe more intimately linked with the underlying pituitary tumor histopathology.

#### IGF-1 normalization

To better delineate the relationship between glucose status and underlying tumor pathology we next examined the impact of glucose metabolism and IGF-1 normalization to assess possible interactions.

#### IGF-1 normalization, glucose status and pituitary tumor size

**Figure 3:**
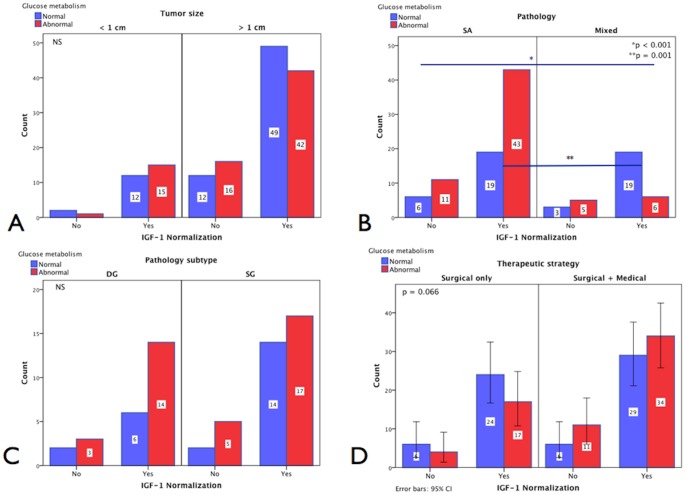
Glucose metabolism and IGF-1 normalization. A. IGF-1 and glucose metabolism according to pituitary tumor size. From the group of patients with microadenomas (n = 30), 12 [40.0%] cases with normal and 15 [50.0%] with abnormal glucose metabolism reached IGF-1 normalization compared with 2 [6.5%] with normal and 1 (3.2%) with abnormal metabolism who did not reach IGF-1 normalization (NS); whereas in the macroadenomas group, 49 [41.5%] with normal and 42 [35.6%] with abnormal glucose metabolism normalized IGF-1 vs. 12 [38.7%] with normal and 16 [51.6%] with abnormal glucose metabolism did not **[N = 149]** (NS). **B. IGF-1 and glucose metabolism according to pituitary pathology.** Six [24.0%] somatotroph adenomas showed normal vs. 11 [44.0%] abnormal glucose metabolism and 3 [12.0%] mixed adenomas showed normal vs. 5 [20.0%] abnormal glucose metabolism in the group not reaching IGF-1 normalization (NS) whereas 19 [21.8%] pure SA showed normal vs. 43 [49.4%] abnormal glucose metabolism and 19 [21.8%] mixed adenomas showed normal vs. 6 [6.9%] abnormal glucose metabolism in the group achieving IGF-1 normalization **[N = 112]** (overall: p<0.001). **C. IGF-1 and glucose metabolism according to pituitary pathology subtype.** Two [3.2%] DG adenomas with normal and 3 [4.7%] with abnormal glucose metabolism vs. 2 [3.2%] SG adenomas with normal and 5 [7.9%] with abnormal glucose metabolism remained with abnormal IGF-1 (NS) whereas six [9.5%] DG adenomas with normal and 14 [22.2%] with abnormal glucose metabolism vs. 14 [22.2%] SG adenomas with normal and 17 [26.9%] with abnormal glucose metabolism reached IGF-1 normalization at last visit **[N = 63]** (NS). **D.** **IGF-1 and glucose metabolism according to therapeutic** **strategy**. In the surgical group, 24 [47.1%] of cases with normal and 17 [33.3%] cases with abnormal glucose metabolism normalized IGF-1 vs. 6 [11.8%] normal and 4 [7.8%] abnormal who remained with elevated IGF-1 (NS). In the surgical+medical group, 29 [36.2%] normal and 34 [42.5%] abnormal normalized IGF-1 vs. 6 [7.5%] normal and 11 [13.8%] abnormal who did not reach IGF-1 normalization **[N = 148]** (NS).

From 149 cases with evaluable information, in the group of patients with microadenomas, IGF-1 normalization was reached by 12/30 [40.0%] with normal and 15/30 [50%] with abnormal glucose metabolism. In contrast, 2/30 [6.5%] with normal and 1/30 [3.3%] with abnormal glucose metabolism did not reach IGF-1 normalization (NS). In the macroadenoma group, IGF-1 normalization was reached by 49/119 [41.5%] with normal and 42/119 [35.3%] with abnormal glucose metabolism compared to 12/119 [10.1%] with normal and 16/119 [13.4%] with abnormal glucose metabolism who did not **[N = 149]** (NS) [Figure 3–A].

#### IGF-1 normalization, glucose status and pituitary pathology

From 112 evaluable cases with complete pituitary histopathology, pure SA was identified in 6/25 [24.0%] with normal and in 11/25 [44.0%] with abnormal glucose metabolism in the group non-normalizing IGF-1 (n = 25), whereas mixed adenomas were diagnosed in 3/25 [12.0%] with normal and 5/25 [20.0%] with abnormal glucose metabolism (NS). In the group reaching IGF-1 normalization (n = 87), patients with pure SA presented with abnormal glucose metabolism more frequently 43/87 vs. 19/87 [49.4% vs. 21.8%] than those with mixed adenomas [p = 0.001] **[N = 112]** (overall comparison: p<0.001) [Figure 3–B]. Additional risk-estimates yielded an odds ratio (OR) of 2.766 (95% CI: 1.490–5.136) for the mixed adenoma group reaching remission; i.e. they are more likely to remit than those with pure SA, particularly those with abnormal glucose tolerance; albeit the latter did not reach statistical significance).

#### IGF-1 normalization, glucose status and therapeutic strategy

Complete therapeutic information was evaluable in 148 cases who underwent pituitary surgery, surgery+medical therapy, and any therapy+radiotherapy. In the surgical group (n = 51), 24/51 [47.1%] cases with normal and 17/51 [33.3%] cases with abnormal glucose metabolism reached normal IGF-1 at last visit whereas 6/51 [11.8%] cases with normal and 4 [7.8%] with abnormal glucose metabolism continued to experience elevated IGF-1 levels (NS). In the group treated with combined surgical and medical therapies (n = 80), 29/80 [36.2%] cases with normal and 34/80 [42.5%] cases with abnormal glucose metabolism achieved IGF-1 normalization compared to 6/80 [7.5%] cases with normal and 11/80 [13.8%] cases with abnormal glucose metabolism who continued to experience an elevated IGF-1 **[N = 148]** (p = 0.066) [Figure 3–D].

For cases treated with surgical+medical approaches, the OR for remission in those with mixed adenomas was 5.982 (95% CI 1.919–18.739). When considering normal over abnormal glucose metabolism in the same scenario, the OR for mixed adenomas was 4.100 (95% CI 1.631–10.304).

### Glucose Metabolism at Baseline and Diabetes at Last Visit

Finally, we compared the proportion of patients with abnormal glucose metabolism at the time of acromegaly diagnosis with final metabolic outcome. The majority of cases showed improvement of their glucose metabolism: 20 [35.1%] cases with abnormal glucose metabolism at diagnosis normalized this parameter whereas 1 [1.8%] normal and 9 [15.8%] abnormal at diagnosis were diabetic after treatment **[N = 57]** (p = 0.012) [Figure S3–A]. Importantly, however, the metabolic glucose phenotype was closely coupled with pituitary histopathology. Specifically, 16/32 [50%] of patients with pure SA had abnormal glucose metabolism at baseline vs. 10/32 [31.2%] normal among those not diabetic at last visit. In contrast, 6/32 [18.8%] of those with abnormal metabolism at baseline remained diabetic at outcome (NS). In patients with mixed adenomas, 1/10 [10%] case with abnormal and 8/10 [80%] cases with normal glucose metabolism at baseline were non-diabetic at outcome whereas only 1/10 [10%] was diabetic at last visit and had abnormal glucose metabolism at diagnosis (NS) **(N = 42)**; [The overall comparison pure SA vs. mixed adenomas was statistically significant: p = 0.013] [Figure S3–B].

## Discussion

More than 75% of 269 acromegalic patients achieved IGF-1 normalization consistent with the effectiveness of current therapeutic approaches for managing this disease. Although several end-points have been proposed to assess disease remission [18]–[20], IGF-1 normalization has emerged as a valuable tool [15], [21], [22]. In this regard our study focused on clinic-pathologic features that portend IGF-1 normalization.

As expected, our analyses revealed more successful IGF-1 normalization in patients with smaller tumors although this was of borderline significance [23], [24]. Nevertheless, pituitary tumor size or radiographic invasiveness did not reveal a significant association with abnormal glucose metabolism at presentation. Instead, nearly 50% of patients with pure somatotroph adenomas displayed abnormal glucose metabolism as opposed to only 10% of those with mixed adenomas. This represents a significant association of pathology diagnosis with abnormal glucose metabolism at the time of diagnosis that was independent of pituitary tumor size, invasiveness, or presenting GH or IGF-1 levels.

We next examined the interactions between glucose metabolism at diagnosis and IGF-1 normalization. The micro and macroadenoma groups displayed similar proportions of patients with normal and abnormal glucose metabolism achieving IGF-1 normalization. Higher proportions of cases with pure SA were noted to have abnormal glucose metabolism compared to those with mixed adenomas. Higher proportions of cases, both in the normalizing and non-normalizing IGF-1 groups occurred with SG compared to DG but this did not reach statistical significance, most likely due to the small number of cases with complete pathology sub-typing.

Similar proportions of normal and abnormal glucose metabolism were observed in both the IGF-1 normalizing and non-normalizing cases in the surgical group. In the combined surgical and medical therapy group, the proportions of normal and abnormal glucose metabolism were also comparable. Between therapeutic strategies, the combined group clearly showed higher proportion of abnormal glucose metabolism for both IGF-1 groups, attaining only borderline significance.

Our study also shows that patients with pure SA constitute the majority of those with pre-existing diabetes noted already at the time of acromegaly diagnosis. Patients with abnormal glucose metabolism tended to require more combined therapeutic interventions than surgery alone. This is consistent with the notion that those with previous diabetes, and most probably pure SA, required more therapeutic interventions. Moreover, those with pure SA were less likely to normalize IGF-1 than those with mixed adenomas. Patients receiving octreotide alone as medical therapy attained IGF-1 normalization with similar proportions independent of glucose metabolism. Importantly, however, twice as many cases with abnormal glucose metabolism at presentation required adjuvant medical treatment consistent with a more severe disease phenotype that requires more interventions to achieve acromegaly control.

Our group has previously described differences in response to treatment, according to pathology phenotypes, both for combined surgical and medical management [1] and SSA alone [15]. In this study we uncovered metabolic differences to the pathology phenotype in describing a distinct disease entity. Although we did not find significance for somatotroph subtypes, patients with pure somatotroph adenomas as opposed to the mixed adenomas displayed a significantly higher proportion of abnormal glucose metabolism and required more interventions to achieve acromegaly control.

It is important to note that serum PRL levels was not informative regarding altered glucose metabolism or pituitary pathology in our populations. Our findings are in contrast to some earlier reports describing hyperprolactinemia as a predisposing factor with increased risk of metabolic syndrome [25], [26]. Nonetheless, our results are more in-line with more recent findings showing that GH-secreting adenomas are more often associated with diabetes than GH- and PRL-secreting ones [27]. In the latter study however, the association was found only with serum hormonal levels and was not corroborated with pituitary pathology findings. These studies support a more relevant role of GH/IGF-1 than PRL in the development of glucose intolerance/diabetes. In vitro and in vivo studies also support a differential involvement of GH and PRL at adipose tissue level mediated by secretion and receptor regulation of adiponectins [28]. Our findings also point to the importance of detailed pituitary pathology in managing patients with acromegaly. Moreover, the use of polyclonal antisera that can result in cross-reactivities between GH and PRL can provide misleading histopathologic diagnoses [17], [23]. The specific detection of PRL immunoreactivity is important however, while mammosomatotrophs and mixed adenomas are clearly different entities, they are functionally distinct as a group from pure somatotroph adenomas [23]. Other groups had previously described prevalences of 20 to 28% for abnormal glucose metabolism in acromegaly cohorts, mainly associated with increasing age and disease progression [29], [30]. Nonetheless, these features were not previously examined in the context of pituitary histopathologic findings.

The relationship between the hormone excess of acromegaly and its complications has been widely studied. Such is the case of colon polyps; nevertheless, several studies have failed to show a strict relationship between colon polyps and the degree of GH/IGF-1 hormone levels [6]. These earlier findings and those shown here provide emerging examples of hormone-independent acromegaly-associated co-morbidities. We speculate that common genetic events may underlie the pathogenesis of acromegaly and its co-morbidities. For example, we recently showed that a germline polymorphism in the fibroblast growth factor receptor 4 (FGFR4) can facilitate pituitary somatotroph tumorigenesis [31]. This same genetic polymorphism has also been shown to play a role in colon cancer growth and progression [32].

Impaired glucose metabolism is a widely recognized complication associated with acromegaly and its other co-morbidities [5], [7], [22], [33]. It represents a critical component of the screening and surveillance plans for such patients. Moreover, interventional studies have shown that acromegaly control and normalization of GH levels are associated with restoration of glucose metabolism [10]. In particular, improvements in metabolic glucose control appear to be more successful with the use of GH receptor antagonism [11]. Here, we show that other non-hormonal factors may play a role in governing glucose homeostasis in acromegalic patients. Indeed, the same aforementioned FGFR4 genetic polymorphism has recently been described to influence pancreatic islet function and the risk of diabetes [34].

In conclusion, we describe here glucose metabolism as part of a distinct clinic-pathologic syndrome. Specifically, patients with pure somatotroph adenomas -as opposed to those with mixed hormone composition- are more likely to present with glucose impairment. Additionally, patients with pure SA are less likely to remit despite the same therapeutic strategies. Our findings raise the question of a hormone-independent common mechanism underlying the pathogenesis of diabetes at least in a subset of patients with acromegaly.

## Supporting Information

Figure S1 **Pre-surgical IGF-1 according to: A. pituitary tumor size, B. glucose metabolism and remission and, C. glucose metabolism and pituitary tumor size.**
(TIFF)

Figure S2 **Growth hormone levels at presentation.** Random GH (rGH) and nadir GH (nGH) according to: A. IGF-1 normalization, B. pituitary tumor size, C. glucose metabolism status at presentation.
(TIFF)

Figure S3 **Glucose metabolism at baseline and diabetes at last visit.** In A, 20 [35.1%] cases with abnormal glucose metabolism at diagnosis normalized this parameter whereas 1 [1.8%] normal and 9 [15.8%] abnormal at diagnosis were diabetic after treatment [N = 57] (p = 0.012). In B, glucose metabolism at baseline and diabetes at last visit according to pathology: 16/32 [50%] of patients with pure SA had abnormal glucose metabolism at baseline vs. 10/32 [31.2%] normal among those not diabetic at last visit. In contrast, 6/32 [18.8%] of those with abnormal metabolism at baseline remained diabetic at outcome (NS). In patients with mixed adenomas, 1/10 [10%] case with abnormal and 8/10 [80%] cases with normal glucose metabolism at baseline were non-diabetic at outcome whereas only 1/10 [10%] was diabetic at last visit and had abnormal glucose metabolism at diagnosis (NS) (N = 42); [Overall comparison SA vs. mixed adenomas: p = 0.013].
(TIFF)
