# Supplementary figures and images for: Metabolic Glucose Status and Pituitary Pathology Portend Therapeutic Outcomes in Acromegaly
Source: PLoS One. 2013 Sep 9;8(9):e73543. doi: 10.1371/journal.pone.0073543 (PMC3767813; doi:10.1371/journal.pone.0073543)

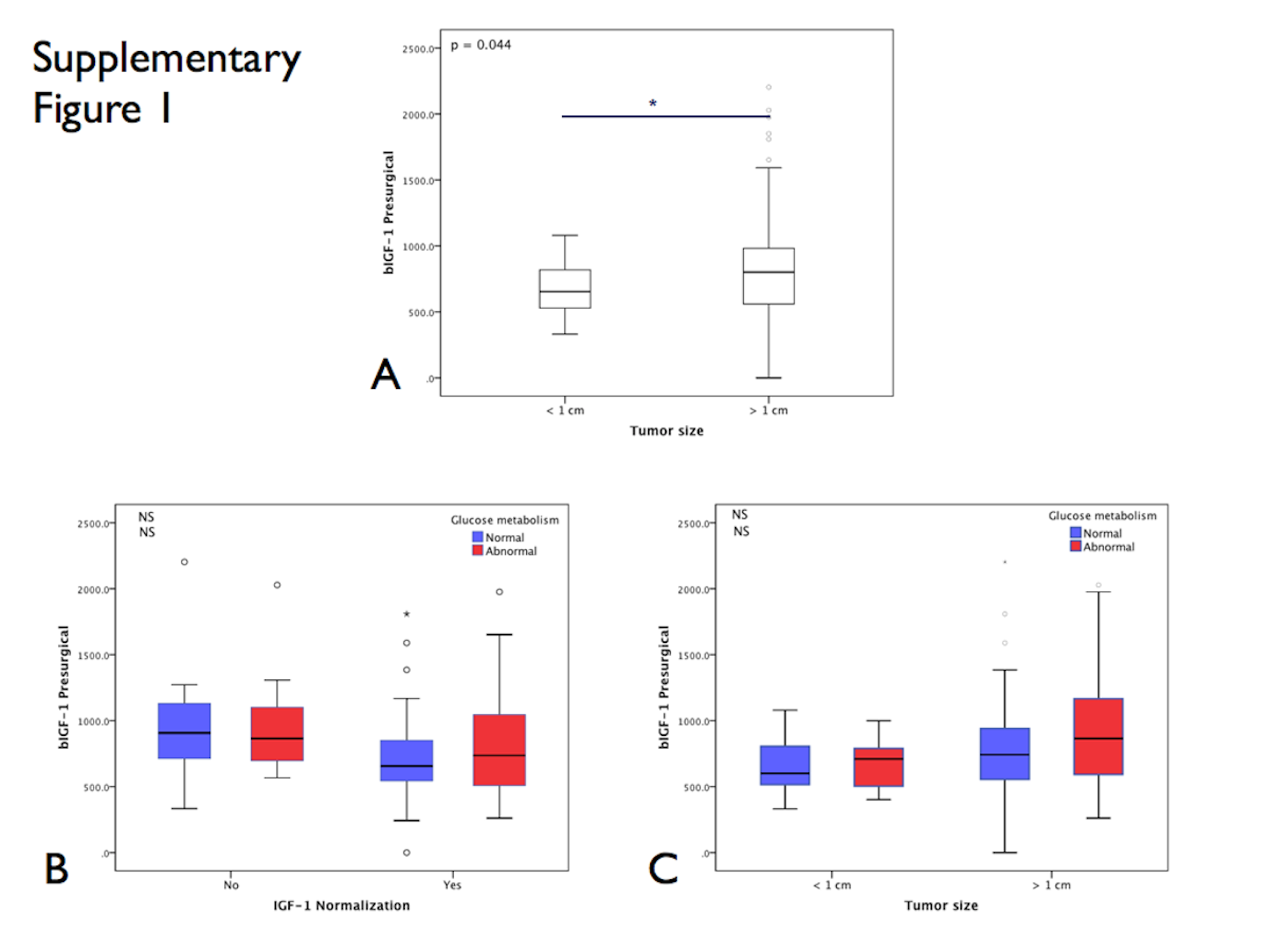

Supplement: Figure S1 — Pre-surgical IGF-1 according to: A. pituitary tumor size, B. glucose metabolism and remission and, C. glucose metabolism and pituitary tumor size. (TIFF) [file pone.0073543.s001.tiff]

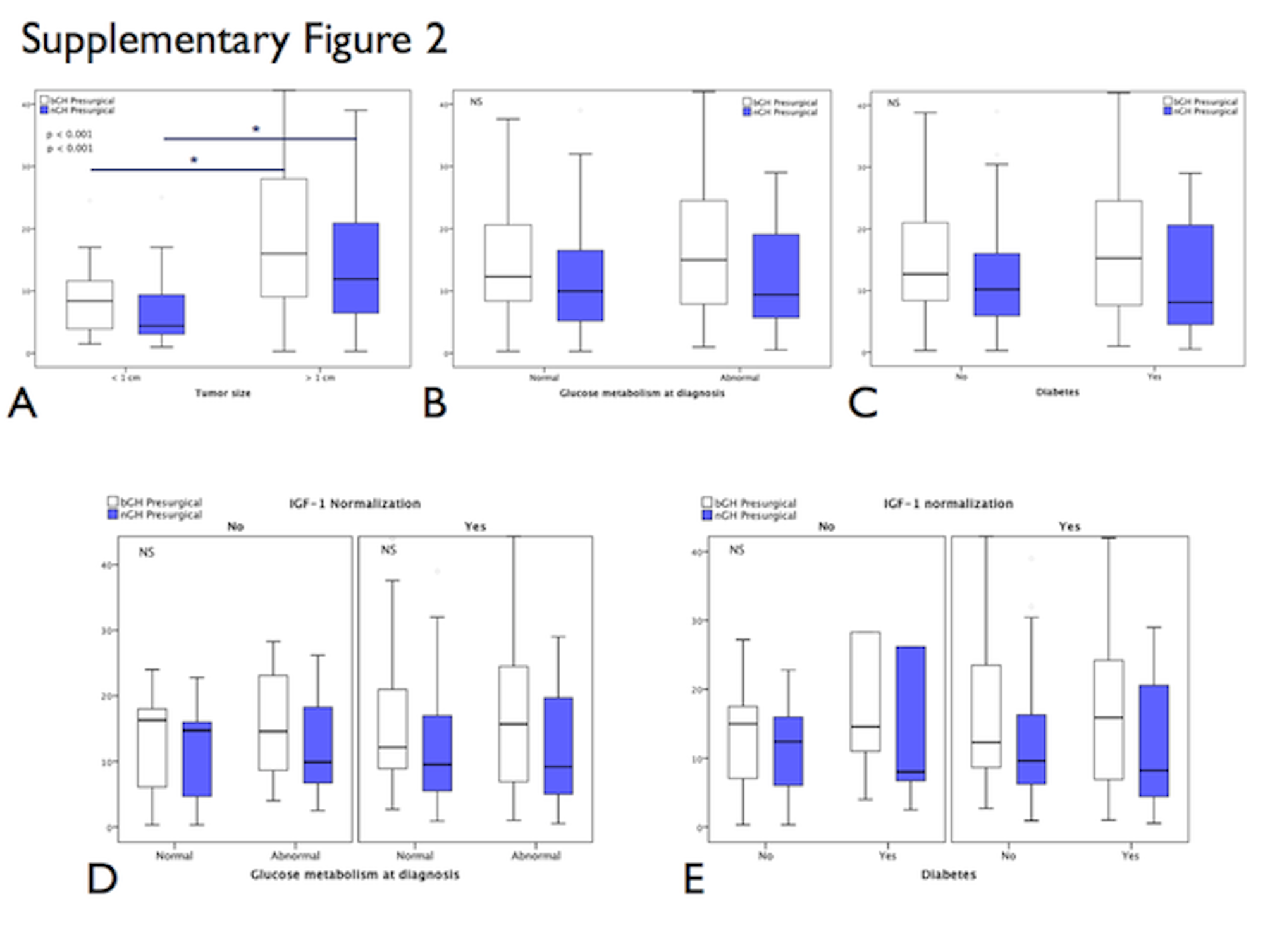

Supplement: Figure S2 — Growth hormone levels at presentation. Random GH (rGH) and nadir GH (nGH) according to: A. IGF-1 normalization, B. pituitary tumor size, C. glucose metabolism status at presentation. (TIFF) [file pone.0073543.s002.tiff]

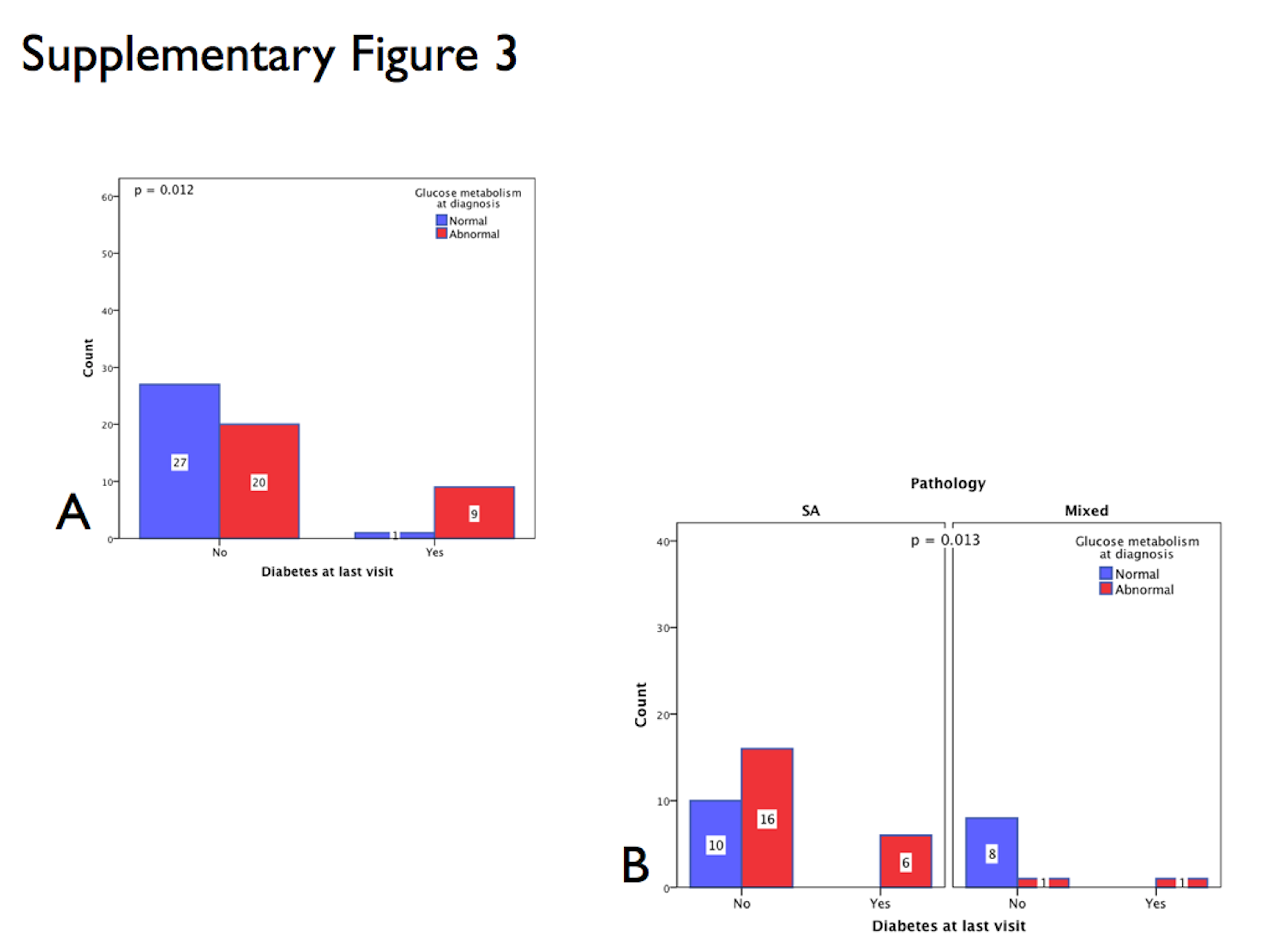

Supplement: Figure S3 — Glucose metabolism at baseline and diabetes at last visit. In A, 20 [35.1%] cases with abnormal glucose metabolism at diagnosis normalized this parameter whereas 1 [1.8%] normal and 9 [15.8%] abnormal at diagnosis were diabetic after treatment [N = 57] (p = 0.012). In B, glucose metabolism at baseline and diabetes at last visit according to pathology: 16/32 [50%] of patients with pure SA had abnormal glucose metabolism at baseline vs. 10/32 [31.2%] normal among those not diabetic at last visit. In contrast, 6/32 [18.8%] of those with abnormal metabolism at baseline remained diabetic at outcome (NS). In patients with mixed adenomas, 1/10 [10%] case with abnormal and 8/10 [80%] cases with normal glucose metabolism at baseline were non-diabetic at outcome whereas only 1/10 [10%] was diabetic at last visit and had abnormal glucose metabolism at diagnosis (NS) (N = 42); [Overall comparison SA vs. mixed adenomas: p = 0.013]. (TIFF) [file pone.0073543.s003.tiff]
